# Facilitators and Barriers to Health Seeking among People Who Use Drugs in the Sunyani Municipality of Ghana: An Exploratory Study

**DOI:** 10.1155/2021/2868953

**Published:** 2021-08-21

**Authors:** Abdul Cadri, Bonyo Abdul Aziz Nagumsi, Alberta Twi-Yeboah, Linda Darko Yeboah, Augustine Adomah-Afari, Maria Goretti Ane-Loglo, Richard Gyan Aboagye

**Affiliations:** ^1^Department of Social and Behavioural Science, School of Public Health, College of Health Sciences, University of Ghana, Legon, Accra, Ghana; ^2^Department of Health Policy, Planning and Management, School of Public Health, College of Health Sciences, University of Ghana, Legon, Accra, Ghana; ^3^School of Law, University of Ghana, Legon, Ghana; ^4^West Africa Drug Policy Network, East Legon, Accra, Ghana; ^5^School of Public Health, University of Health and Allied Sciences, Ho, Ghana

## Abstract

Drug use is one of the global public health issues, and its accompanying disorders have consequences on people's mental, physical, and environmental health. Nevertheless, the majority of people who use drugs have never been treated for drug dependence and other health conditions whilst others discontinue their treatment for drug use disorder. Using the health belief model, the study aimed at exploring facilitators and barriers to health-seeking among people who use drugs in the Sunyani Municipality of Ghana. A descriptive study design was used, employing a qualitative approach. In-depth interviews were conducted with a total of 22 participants, including two key informants (male and female). The first group of participants was recruited from the ghetto (an area in the municipality where people who use drugs are usually located). The other group of participants was recruited using hospital-based records. The interview data were transcribed, coded, and analysed for the generation of themes with the aid of Nvivo version 12 pro. The results showed that people who use drugs face health challenges such as drug dependence, malaria, lungs and breathing complications, cardiovascular complications, and skin complications. People who use drugs experienced poor perceived quality of life and low health status. Health-seeking behaviours of interviewees were influenced by the perceived benefit, perceived severity, cues to action, among others. Multiple sources of healthcare were used by the people who use drugs. Whereas ease of communication, perceived severity, benefit, among others were facilitators to their health-seeking behaviours, cost, dwindling social support, lack of knowledge of the condition, and fear of arrest by law enforcement agencies also served as barriers to seeking healthcare at the orthodox health facilities. This paper suggests a holistic approach to help improve the health and health-seeking behaviours of people who use drugs. The researchers wish to indicate that an earlier version of this manuscript has been presented at the University of Ghana as a thesis.

## 1. Introduction

Drug use is a global public health issue that transcends cultural boundaries; it is not an issue that is related to a specific cultural group, race, or people from a specific geographical location [1]. It is prevalent in most parts of the world and Ghana is not an exception. In Ghana, a study reported the daily use of alcohol and marijuana among the youth to be 12% and 16.2% respectively [2]. Another study further indicated that about 5.3% of the Ghanaian school-going population reported a past-month use of marijuana whilst 7.1% reported a lifetime use of amphetamine [3].

Illicit drug use is a serious public health issue with a tremendous health and economic impact [4, 5], which is usually accompanied by high comorbidity between drug dependence, medical and mental disorders, and a host of social problems such as homelessness, criminal justice involvement, unemployment, and financial constraints leading to poverty in some cases [6]. Several substances or drugs (alcohol, cocaine, crack, etc.) are being used; each with its own accompanying side effects, health, and social problems [7]. Other studies reported that drug dependence is associated with poor dental health and skin complications such as spider telangiectasias, jaundice, pruritus, hyperpigmentation, skin ulceration, and psoriasis [8, 9]. The physical, mental, and social health needs of people who use drugs have been documented worldwide [6, 8, 10, 11].

Several factors influence the health-seeking behaviour among people who use drugs, including one's desire to solve their own problem, perceived need, health literacy, and financial capability [12]. It has been established that literacy (knowledge and beliefs that aid in recognising, managing, and preventing mental health-related issues) and different ways of conceptualising the issue have a great influence on the health-seeking behaviour among people who use drugs [13].

Most at times, for people to seek healthcare, they need to consider the symptoms of the ailment as a threat to their health and have the required resources at their disposal [14]. A study reported that majority of the respondents (40.0%) did not know where to go for treatment for drug dependence, 30% thought that treatment was too expensive, 17.5% that treatment was not effective, and only 15% revealed other causes [15].

A report estimated that 1.25 million Ghanaians were having problems with drug use, mostly marijuana, whilst others listed were cocaine, heroin, methamphetamines, and other synthetic opioids such as tramadol and codeine [16]. Another report indicated that drug use was on the rise in the Sunyani Municipality as well as incidence of drug use related disorders noting that 26% (596) of 2284 patients who utilised the mental health facility within the municipality were alcohol and drug related cases [17, 18]. These statistics showed a 12% increase in substance use and relapse cases, compared to that of the preceding year.

Despite the documented evidence of the effects of illicit drug use on the individual, family, and nation as a whole, many people who use drugs do not access healthcare services [6, 10]. Although the problem exists in Ghana, there appears to be limited studies conducted on the facilitators and barriers to health-seeking behaviours among people who use drugs in the Sunyani Municipality. This study sought to fill the gaps in literature accordingly.

## 2. Theoretical Perspective

The concept of health-seeking behaviour has been defined as any action taken by individuals who perceive themselves to be suffering from a health problem or illness with the aim of finding a remedy to the condition [19]. The Health Belief Model (HBM) was adopted at the start of the study to help identify the health-seeking behaviours of respondents at the individual level. The HBM is based on a person's belief to take a health-related action if that person: feels that a negative health condition can be avoided; has a positive expectation that by taking a recommended action a negative health condition could be avoided; and believes that can successfully take a recommended health action [20]. A major strength of the model is its applicability in determining why people do and do not access a health intervention, as well as it is ideal for planning educational interventions. For this study, HBM was appropriate because it is important that we know why people who use drugs do do not access health services, which willinfluence designing an intervention to improve access to health services among people who use drugs.

The model proposed that a health-related behaviour is influenced by an individual's perception of six key tenets; perceived susceptibility, perceived severity, perceived benefits, barriers, cues to action, and self-efficacy [13]. The HBM proposed that individuals faced with alternatives in taking actions may choose the one that is most likely to yield a positive outcome [21].

In applying these constructs in this study which focused on the case of people who use drugs, it was theorised that the perceived susceptibility to a particular health problem and the perceived seriousness of that health problem would influence their health-seeking behaviour. The perceived susceptibility is the situation where people who use drugs have the belief that they have a chance of suffering from a particular condition and the perceived seriousness/severity of the health problem is the situation where they see how serious a health problem is and its accompanying consequences [19]. Additionally, Cues to action, which basically constitutes the strategies to activate readiness such as witnessing the death of a colleague of critical health condition also have an influence on an individual's health-seeking behaviour [19]. Moreover, the likelihood of a person who uses drugs taking an action is influenced by the perceived benefit of the action as against the barriers to taking a particular action and the likelihood of a behavioural change. These likelihoods of an action are influenced by the factors mentioned above. Therefore, the HBM was used to explain the relation between a person who uses drugs' belief and health behaviour at the individual level by giving an understanding of their health behaviour.

## 3. Materials and Methods

### 3.1. Study Design

A descriptive study design was used, employing a qualitative research approach, specifically, phenomenological study. Based on the objectives of this study, the researchers applied the constructivist research paradigm [22].

### 3.2. Study Setting

The study population constituted people who use drugs, residing in the Sunyani East Municipality in the Bono Region of Ghana. According to the 2010 population and housing census [23], it has a total population of 123,224 (61,610 males and 61,614 females). It has a land area of 506.7 km^2^. It lies between latitudes 7°20′N and 7°05′N and longitudes 2°30′W and 2°10′W. It shares border with Sunyani West District, Dormaa East District, Asutifi District, and Tano North District.

According to Danso-Appiah, the delivery of healthcare in Ghana is based on the concept of primary healthcare [14]. In each district capital, at least one government hospital is located and staffed by one or more qualified medical doctors, nurses, pharmacists, laboratory technicians, auxiliary nurses, and other support staff. District hospitals deal with all cases except specialized care and serious cases, which are referred to the regional tertiary hospitals. It is indicated that the main sources of finance for public healthcare institution in Ghana are government subvention, internally generated funds, and donor-pooled funds [24]. The National Health Insurance Scheme (NHIS) has come to replace the out-of-pocket payment system for healthcare delivery in public health facilities in Ghana and has been the primary method of payment for subscribers [25]. Nevertheless, it is reported that some facilities charge user fees while others have reintroduced the out-of-pocket payment system due to the inability of the government to reimburse service providers for services rendered to NHIS subscribers [26].

### 3.3. Participant's Recruitment

A total of 22 participants were recruited: with two of them being key informants. One of the key informants was a female psychiatric nurse who has been providing healthcare for people who use drugs and had specialist knowledge of them. The other key informant was from a ghetto (a specific area where people who use drugs are usually based) and was a current person using drugs with more than 10 years experience in drug use and was considered their leader. Their inclusion in the study improved trust and they provided expert opinions on the subject matter. Participants were recruited from both the hospital and the ghetto. The hospital consisted of people who use drugs and had been diagnosed of drug dependence but still used the drug and the participants from the ghetto consisted of people who use drugs and had never been diagnosed of drug dependence according to the International Classification of Diseases (ICD) 10 (WHO,[27]).

The snowballing method was used to sample participants from the ghetto and purposive sampling was used to sample respondents from the hospital. The key recruitment criterion was that the participant still uses drugs at the time of the study. Other inclusion criteria were that participants should be between the ages of 18 and 65 years, had used drugs at least once in the past one month, and were in a sober state and not ill at the time of data collection. Recruitment of participants from the hospital was done by contacting the head of the mental health unit who used hospital records to identify service users who met the inclusion criteria. Participants from the ghetto were recruited by first contacting a gatekeeper. The gatekeeper was informed about the study, and he led the interviewers to the area they were usually located. They were informed about the study and its inclusion criteria. Participants who met the inclusion criteria and consented were then recruited for the study.

### 3.4. Data Collection

In-depth interviews were organised for interviewees at their convenient location and time without interruption, after dialoguing with them to arrive at a consensus. Interviews were conducted with the use of semistructured interview guides developed by the authors. The interviews were conducted by the principal investigator and a female researcher (ATY) who are both social and behavioural scientists. A third researcher (BAAN) also assisted in the interviews. All interviews were conducted in a Ghanaian local language (Asante Twi). The interviewers had intense training in qualitative research at the Social and Behavioural Science Department of the School of Public Health at the University of Ghana, Legon. They had postgraduate degrees in Public Health with experience in qualitative research.

An interviewer guide was used to obtain data from the participants on key areas such as sociodemographic characteristics, their healthcare needs, illness characteristics, and their health-seeking behaviour and practices. Another guide focused on key informants' views of participants' health needs, their health behaviours, and factors influencing continuity of drug use in the Sunyani Municipality. More so, the barriers to health seeking wereexplored from the perspectives of both the key informants and the participants.

The semistructured interview guides were developed in English and translated into the local language (Asante Twi) by language experts using a back-to-back strategy. This strategy ensured that a language expert proficient in English and the local language translated the interview guides from English to the local language and another expert translated it from the local language to English. The two conversions were then checked, and where there was a difference, it was discussed among the language experts with a third language expert as the mediator. This was done to ensure uniformity in data collection tools. Participants were sampled until saturation was reached and no new information was achieved [44]. The interview guides were pilot tested using three participants. All ambiguous questions were reworded, and the necessary adjustments were made. The participants for the pilot study were not included in the data collection for the main study. No repeat interviews were carried out.

The in-depth and key informant interviews were digitally recorded and transcribed verbatim lasted for about 25 to 40 minutes each. Field notes were made promptly after each interview and transformed into a data document. Participants were given a compensation for their time in the form of a Vodafone or MTN recharge card.

### 3.5. Data Analysis

The tape-recorded interviews were transcribed by two members (ATY, BAAN) of the research team independently and compared for consistency. Any inconsistency was discussed among them with a third member (CA) as a mediator. Transcripts were anonymised by assigning unique codes to protect the identity of the participants. Each transcript was checked for accuracy against the original recordings before they were analysed. The transcripts were read all over again to gain a broader understanding of participants' health-seeking behaviours before they were imported into NVivo 12 pro software for analysis. The analysis was done independently by two members (ATY, BAAN) of the research team, and consensus was formed on the emergence of superordinate and subordinate themes with the first author (CA). A thematic analysis was used, employing both deductive and inductive analysis [30].

A codebook was then generated. Each transcript was opened in the NVivo software, and line-by-line reading and coding of all the statements were done. The coding was reviewed, where some categories were developed and merged to develop themes. GT1-GT14 represented interviewees from the ghetto. HB1-HB6 represented hospital-based interviewees. KII1-KII2 represented key informant interviewees.

The ethical considerations in this study including information about the research, privacy and confidentiality, voluntary participation and withdrawal, risks and benefits, and results dissemination were all made known to the participants before interviewing them. These were contained in the participant's consent form, which was approved by the Ethical Review Committee of the Ghana Health Service Research and Development Division in Accra with reference number GHS-ERC 056/03/19. The study procedures and all necessary information were well explained to the participants. Those who agreed to participate in the study were given a written consent form to sign (if literate) or thumbprint in the presence of a literate witness (if illiterate). Formal permission was granted by the Sunyani Municipal Health Directorate, as well as the gatekeepers of the health facility and the ghetto. Participants had no prior relationship with the interviewers/researchers but were made aware that the interviewers were social and behavioural scientists who had a background in public health. Permission to audio record interviews was sought from participants before the interviews commenced.

### 3.6. Quality and Trustworthiness

Quality and trustworthiness are important components of qualitative research. To ensure the quality and trustworthiness of this study, criteria outlined by Lincoln and Guba [28] were followed in this study; they include credibility, transferability, dependability, and confirmability. To ensure the credibility of this study, the researchers spent enough time on the field to study the phenomenon of interest, as well we adopted the method of triangulation. A peer was also allowed to review the work, and a few of the participants were contacted again to ascertain that information captured was a true reflection of their experience. To ensure the transferability of this study, the researchers have provided detailed information on the procedures and steps taken in the research for replication and application in other contexts and domains. In ensuring the dependability of this study, we used external audits, where a researcher who was not part of the study was made to assess this study. Lastly, to ensure confirmability, the study included multiple investigators and also acknowledged the limitations of the study.

## 4. Results

### 4.1. Participants' Characteristics

A total of 22 interviewees sampled from the ghetto and the hospital-based records participated in the study (including 2 key informants). The age of participants sampled from the ghetto ranged from 22 to 60 years, and those from the hospital-based records were between 31 and 45 years. The ages of the key informants from the ghetto and the hospital were 37 (male) and 36 (female nurse) years, respectively. The majority of the participants were Christians with some level of education. Most of the participants from the ghetto were unemployed while most of the participants sampled from the hospital were employed. A very few of the respondents were married with the majority being either single or divorced. These have been shown in Table 1.

### 4.2. Healthcare Needs of People Who Use Drugs

It could be argued epidemiologically that, since people use drugs on a regular basis, they may be exposed to certain health conditions, which this study sought to establish.

#### 4.2.1. Malaria

The findings of the study showed that malaria and its accompanying symptoms like body weakness and headache were one of the predominant health challenges among interviewees.

*“The main health challenge we the junkies face is malaria, because we are down here (ghetto) with the mosquitoes… They always bite us and later lead to malaria but when the ‘turkey' (withdrawal symptoms) also comes, then it brings all those sicknesses… But when you get some of the cocaine, you get a bit okay and covers the malaria… It makes you forget the malaria… But the moment the effect of the cocaine wears off, then the sickness comes again…” (KII-1)*.

*“One disease that has been disturbing me a lot is malaria; even right now, I am suffering from malaria…” (GT1)*.

#### 4.2.2. Drug Dependence

The respondents reported symptoms of withdrawal due to drug dependence and indicated that it was a big challenge for them:

“*The way I feel in my body when I don't get some of the drugs is severe than any form of illness, because if I don't use some (drug) the whole day, I can't move, I will just be lying down… Even if they say the police are coming, I will not be able to run and I see that to be a sickness as well…” (GT11).*

#### 4.2.3. Lung Related Complications

Another predominant health challenge faced by the people who use drugs, especially those from the ghetto was lung and breathing complications such as chronic coughing and coughing up black nonbloody phlegm:

*“There is one sickness like pneumonia that affects us… You feel pains in your ribs… Because when you use the drugs for long, it wears off your rib bones… And it also affects your lungs… So, when the lungs become weak, you feel some pain in your rib section when breathing…” (KII-1).*

*“I have been experiencing pains in my ribs… When I cough, the phlegm is even black… It is not that serious though… Sometimes, it comes and goes off…” (GT7).*

#### 4.2.4. Cardiovascular Complications

Four (4) interviewees also reported cardiovascular complications such as abnormal heartbeat or rapid heart rate with other illnesses:

*“I am suffering from HIV and I am on medication… But due to this life (drug use), I don't adhere to the treatment well… I also experience episodes of headache and when I walk, I feel tired and my heart starts beating faster…” (GT9).*

#### 4.2.5. Gastrointestinal Complications

Furthermore, the findings indicated that gastrointestinal complication was a health challenge that was experienced by the participants from the ghetto:

*“…You will feel so severe pain in the stomach and we have to rush you to the hospital...” (KII-1).*

#### 4.2.6. Dermatological Conditions

From the field notes, other health challenges that were observed among interviewees from the ghetto included dry patches on the skin and cracked fingertips.

#### 4.2.7. Oral Health Issues

The field notes also revealed that most of the interviewees had oral health issues such as rotten teeth and some missing teeth. An interviewee further reported this as a challenge:

*“Even my teeth are destroyed…A lot of things have spoilt in my life so there are times I regret” (GT11)*

#### 4.2.8. Health Status

The interviewees from the ghetto reported a poor health status as compared to those from the hospital who reported that their health status was good and they felt healthy. It was also observed that an important social factor that undermines the health of most of the interviewees from the ghetto was homelessness:

*“I am not healthy… Not healthy at all…” (GT9).*

*“For now, my health status is very good… Previously, it was bad but after I visited the hospital, it is good now…” (HB2).*

### 4.3. Facilitators to Health Seeking

The interview data revealed two subthemes, which have been categorized into health-seeking practices and determinants of seeking illness care. The subtheme on health-seeking practices further constituted the drug users' illness perception, illness identification, and their strategies to mitigate illness.

### 4.4. Health Seeking Practices

Findings showed that interviewees from the ghetto had a common perception of illnesses they usually experienced. It was shown that they perceived illness as withdrawal symptoms, which they called “turkey” due to drug dependence:

*“Whenever I feel sick, I see it to be ‘turkey'… So, when I get some of the drugs to use, then it stops… But after some few minutes that the effect of the drug wears off, then those symptoms come back… So, it doesn't make me feel that it's sickness, I see it to be ‘turkey'… It happened to me at some point in time and I got admitted at the hospital…” (GT12).*

*“I have never been to the hospital… I have a perception that when not feeling well, then its “turkey” …But usually, after using the drug (cocaine), I feel alright...” (GT3).*

Additionally, the findings showed that the interviewees had a similar way of identifying their illnesses. They reported that they were able to identify that they were sick when symptoms of the sickness persisted after using the drugs (mainly cocaine and heroin). In most cases, they first saw their sickness as “turkey” or withdrawal symptoms:

*“Hmm, for now, when I am not feeling well, I see the symptoms to be severe than what I experience when ‘turkeying'… Right now, I have a cold and I thought it was ‘turkey'… When I came, they were all saying it's ‘turkey' but after using the drug, it's still there… Then, I knew it was not the ‘turkey' but I'm sick…” (GT12).*

*“The first thing I think of is, it is because I have not been able to get some of the drug to use… So, after using it (cocaine) and I still feel unwell, then I know that I am sick…” (GT5).*

However, interviewees from the hospital were able to identify it quickly when they were sick:

*“For that one, I am able to see… That is, if any part of my body is aching, I notice it… The main difference is, with this one, it is in the mind… But if it is something like I am visiting the toilet too many times, I can see something is wrong…” (HB1).*

#### 4.4.1. Strategies to Mitigate Illness

The analysis revealed that interviewees had diverse strategies to mitigate illness. They resorted to using different strategies and means of healthcare and sometimes simultaneously use multiple sources of care. Due to the reported illness perception and identification, interviewees from the ghetto mainly resorted to using drugs (mainly cocaine and heroin) as their first treatment source when experiencing symptoms of ill-health:

*“If I will fall sick, then maybe it is something like headache, and the moment I get the cocaine to use, it will be gone…” (GT2).*

*“Whenever I don't feel well, I go to the ghetto…I don't go to the hospital and don't go to any pharmacy… I come straight to the ghetto to get some drugs (cocaine or heroin) to use…” (GT4).*

#### 4.4.2. Hospitals, Clinics, and Local Pharmacy

The use of local pharmacy appeared to be popular among the interviewees from the ghetto, especially when they used the cocaine and still felt symptoms of ill-health:

*“When I'm not feeling well and use the drug and still not feeling well, then I go and buy the medicine from the pharmacy…” (GT10).*

*“I don't go anywhere; I just take the drugs (cocaine or heroin) …Sometimes, I go to the pharmacy to buy medicine, but I use the drugs (cocaine or heroin) more than the medicine that I buy from the pharmacy…” (GT5).*

However, interviewees from the hospital mainly reported to be using the hospital or clinic as their first point of care/call when they were experiencing symptoms of ill-health. Some of them also reported to be using the local pharmacy (over the counter medication):

*“I go to the clinic to get some malaria medication or some of the injection…I used to go to Dr. Asare's clinic but when I went there recently, he said it was because of the alcohol I am drinking, then I came to the government hospital…” (HB1).*

*“Sometimes, I come to the hospital, sometimes, I go to the pharmacy to buy medicine…” (HB3).*

Three of the interviewees also reported using religion and spirituality as a source of treatment sometimes:

*I like to go to the hospital, but when it is not anything serious, I go to the prayer camp to pray… They can give me anointing oil and other things and I get better…” (HB4).*

*Sometimes too, I just fetch water and pray on it and drink it and I will be fine…If you ask a lot of people, they will tell you that I am a wizard” (GT7).*

### 4.5. Determinants of Seeking Care

The findings showed that the determinants of health seeking varied among the interviewees depending on the choice of treatment source. The main determinants that were reported were severity of condition, ease of communication, benefit from action, and readiness to seek care. These have been explained below.

#### 4.5.1. Severity of Condition

One important determinant of illness treatment source was reported to be the severity of illness. Here, the interviewees reported that they took actions based on how severe the illness was, which could influence their decision and illness treatment source:

*“We don't go to the hospital…If someone's condition is serious, we get some paracetamol or amoxicillin or penicillin then buy it for the person…When the person takes them, they get normal a bit…” (KII-1).*

*“I like to go to the hospital, but when it is not anything serious, I go to the prayer camp to pray… They can give me anointing oil and other things and I get better…” (HB4).*

*“I don't think of going right away…I don't usually see it to be serious, so if I try managing it in the house and it doesn't go, then I see it is serious and I take it to the hospital…” (GT8).*

#### 4.5.2. Ease of Communication

The ease at which they were able to communicate with the healthcare provider also influenced their choice of health seeking. For instance, at the pharmacy shop, they indicated that they felt comfortable communicating with the shop attendants and the attendant would give them the medicines they wanted:

*“That is a place that when you go and tell them your sickness and the medicine you use when the sickness comes, they will give it to you for you to treat it…So, when the sickness comes again, then I know these are the medicines I am supposed to take…So, I still have the box of the medicine with the name, when I go to the pharmacy, I just tell them the name of the medicine and they give it to me…” (GT1).*

#### 4.5.3. Benefits from Action

The findings showed that interviewees' health-seeking behaviour or choice of treatment was informed by the benefit that they stood to gain by taking a particular action. This makes the majority of them use more drugs when not feeling well, while others also went to the pharmacy or visited the hospital.

*“I have been coughing, you see right now my voice is not clear…I feel pains within and I am yawning frequently… I feel pains in my calf, but usually, when I get the drug (cocaine), everything vanishes… After the effect of the drug wears off, then it comes back… But as long as I get it to use, I am ok…” (GT5).*

*“When I'm not feeling well and use the drug and still not feeling well, then I go and buy the medicine from the pharmacy…” (GT10).*

*“The best for me is the hospital or a nearby clinic…I see that place to be the best for me…” (HB2).*

#### 4.5.4. Readiness to Seek Care

In addition, the findings showed that there were strategies that activated the readiness of a person who uses drugs to seek care from a particular source. For instance, witnessing the death of a peer and a critical health condition was found to have an influence on a person who uses drugs' health-seeking behaviour. It was reported that they had identified specific critical health conditions that resulted in death if treatment was not sought at the orthodox health facility. Therefore, such conditions made them rush to the hospital to seek treatment:

*“If someone will go to the hospital, then it is one sickness like pneumonia that affects us… You feel pains in your ribs… Because when you use the drugs for long, it wears off your rib bones and it also affects your lungs…So, when the lungs become weak, you feel some pain in your rib section when breathing…So, when it happens and you don't rush to the hospital, you will die…” (KII-1).*

### 4.6. Barriers to Health Seeking

The study also sought to identify specific factors that served as barriers to seeking formal care. Four subthemes emerged, which included the cost of healthcare, dwindling social support system, fear of arrest by law enforcement agencies, and lack of knowledge of the appropriate place to seek healthcare.

#### 4.6.1. Cost of Healthcare

The results of the study showed that, for most interviewees, the main barrier to health seeking at the hospital was the cost involved. Most of them were not patronizing the National Health Insurance and also reported that they did not have the financial resources to cater for the charges involved in seeking treatment at the hospital:

*“I prefer going to the hospital but I don't have health insurance and also I don't have any money on me to pay for the costs involved…” (GT12).*

*“It's because of financial constraints…Maybe, I will come here (hospital) and I might not get money to pay for the cost involved…” (HB3).*

#### 4.6.2. Dwindling Social Support System

The results revealed that the communal spirit (solidarity) which enabled sick family members to be supported by the entire extended family was dwindling. Interviewees who reported to the hospitals for healthcare but could not raise funds to meet the cost of healthcare had to be discharged by healthcare providers:

*“…No, I don't because I don't have money to go to the hospital… Recently, I went to one newly opened hospital and I was referred to the big hospital and I was given a bed there… They asked me which of my family members was coming and no one was there because they were fed up with me… So, when that happened, I wasn't attended to at the hospital and I got off the bed and headed home...” (GT13).*

*“…It's my family that caters for all the charges but now, they are fed up with me...So, even when I go, they don't mind me…This is the time I really want to stop it (drug use) but I don't get help from anyone…” (GT9).*

#### 4.6.3. Lack of Knowledge of the Appropriate Place to Seek Healthcare for Drug Dependence

It is believed that people will take action to seek healthcare if they have information or know of the appropriate place to go for such care. Since the interviewees indicated that drug dependence was a major challenge, they were interviewed on why they did not seek help. The analysis showed that, even though all the people who used drugs reported that they wanted to quit drug use, they were unable to do so because of the challenges associated with the withdrawal symptoms. However, in the midst of this, they do not appear to know where to seek treatment for the drug dependence:

“…*And a lot of people also don't know where to go to in order to be able to stop using the drugs…” (KII-1).*

“*…I have thought about stopping it for long but I don't know where to go to be able to stop…*” (GT6).

#### 4.6.4. Fear of Arrest by Law Enforcement Agencies

It should be emphasised that drugs use is abhorred by many people as well as the law enforcement agencies in the Ghanaian communities. To understand how such societal controls were influencing their healthcare-seeking behaviours, the interviewees were asked to express their opinion on this issue. The analysis showed that fear of being arrested mostly, by the law enforcement agencies like the Police, was a barrier to health seeking at the hospital:

*“Sometimes too, I think about it and get scared that maybe I might be arrested because of the drug use…if not for that, I would have gone there (hospital) long ago…” (GT11).*

## 5. Discussion

The findings of the study have been explained with the theoretical framework of the HBM and related to appropriate literature.

### 5.1. Healthcare Needs of People Who Use Drugs

The study found that the participants faced a lot of health challenges and had diverse healthcare needs, the commonest being drug dependence and malaria. The plausible explanation for the reported finding on malaria could be the geographical location of the ghetto.It was revealed that some of them slept in their base (referred to as ghetto by them), which were surrounded by bushes and stagnant water, which served as breeding places for mosquitoes. Hence, there was likely to be a high rate of malaria among them. This finding was similar to a study in Vietnam which also found that malaria was prevalent among people who use drugs [29].

Drug dependence was found to be a major healthcare need of the people who use drugs in this study. Due to the dependence, people who use drugs always craved for the drugs, and this resulted in withdrawal symptoms anytime they made an attempt to quit. The finding was similar to other studies, which found that drug dependence was a healthcare need among people who use drugs accompanied by withdrawals [30, 31].

Other healthcare needs of the people who use drugs were lung and breathing complications, cardiovascular complications, gastrointestinal complications, dermatological or skin complications, and oral healthcare needs. These findings are in agreement to other studies which reported various complications including cardiovascular, lung, and breathing among people who use drugs [32, 33] [7]; [8, 9, 34].

Almost all the participants from the network of people who use drugs reported low and poor health status and quality of life, which was consistent with the findings of other studies where drug use was associated with a self-reported poor health status and lower perceived quality of life [8, 35]. However, a key addition in our study was that the participants recruited from the hospital reported improved health status and perceived quality of life, indicating the important role of health seeking from the health facility.

### 5.2. Health-Seeking Behaviours among People Who Use Drugs

The findings indicated that people who use drugs adopted diverse health-seeking behaviours and practices. They sought health from different sources, which followed a pattern until the problem was solved. A similar study found that participants resorted to using multiple sources with a pattern of seeking healthcare from a succession of healthcare providers until the problem was solved [21].

It was noticed that most of the interviewees' first source of seeking healthcare when they experienced symptoms of ill-health was to use more drugs. This could plausibly be due to the symptoms of the physical ill-health, which is mostly regarded as withdrawal symptoms and hence, call for more drugs to be used. This finding agrees to the findings by another study that people who use drugs often experience withdrawal symptoms and therefore go in to use more drugs [36]. A key addition is the finding by this study that some interviewees were able to identify that they were suffering from a physical ill-health only after they had used the drug and symptoms of the ill-health still persisted. This practice could lead to a delay in accessing adequate treatment, which could result in tremendous health effects among them and in the worst case, could lead to death.

The use of religion and spirituality was also found to be a health-seeking strategy practiced by some individuals. Some people who use drugs resorted to religion and spirituality when experiencing an ill-health by way of praying and using items such as anointing oil and water to get healed. A study among people who inject performance and image-enhancing drugs also reported the use of religion and spirituality as a health-seeking strategy which corroborates withthe finding of this study [37].

### 5.3. Facilitators to Health-Seeking Behaviours among Drugs Users

Although participants' characteristics such as level of education, income, sex, and marital status were important determinants of health-seeking behaviours, the study focused on service and illness characteristics.

The findings of the study showed that facilitators to health-seeking behaviours and practices varied across individuals but mostly influenced by the illness characteristics and availability of services. The subsequent choice of healthcare was determined by the perceived benefit of treatment, severity of illness, ease of communication with healthcare provider, and cues to action.

The common practice of further drug use as a means of staying healthy or treating symptoms of physical ill-health was motivated by their perceived benefit. This is mainly because symptoms of the ill-health were not felt after using the drugs and experienced it again when the effect of the drug had diminished, and they sought to use more. This practice among interviewees could be influenced by the seeming lack of public health education on specific health conditions that they could face and the appropriate health-seeking practices to adopt [38]. This is similar to findings in other studies which reported that the benefit participants stand to gain by performing a particular health action influences their health-seeking behaviour [21, 39].

The severity of illness was found to be an important determinant of health-seeking behaviours and practices. This was the belief that certain conditions were not serious enough to merit treatment at the appropriate source. This could possibly be due to the low level of education and limited knowledge of the conditions among the interviewees, which could result in a delay in accessing appropriate healthcare, which could affect prognosis negatively. This concept explains their decision on whether or not to take a specific action based on how severe they think the condition is. Other studies conducted in Ghana and Australia also reported that the perceived severity of a health condition was a contributing factor to health-seeking behaviours among their study participants [21, 39].

The ease with which interviewees were able to communicate with healthcare providers was also found to have an influence on the health-seeking behaviour of the people who use drugs. The participants mostly chose the local pharmacy because they were comfortable communicating with the attendants and got the medication of their choice. The concept of ease of communication best explains and predicts this behaviour of people who use drugs with regards to their choice of a health facility. This finding is similar to findings by a study conducted in Ghana that respondents usually use the local pharmacy because it was easy communicating with the medicine sellers at the pharmacy [21]. It was also reported by a study that ease of communication influenced the health-seeking behaviour of respondents [39].

Cues to action also influenced their health-seeking behaviour. Using this as an influencing factor, the interviewees sought healthcare for specific conditions due to past experience or observing a peer suffer or die from that same condition. Therefore, this motivated them to seek formal healthcare when they also experienced similar symptoms or conditions. The concept of cues to action best explains and predicts the behaviour of interviewees seeking healthcare from formal sources on certain conditions that they see causing serious harm to their peers. This finding is supported by findings from other studies conducted in Ghana and Australia where cues to action also influenced the health behaviour of respondents [21, 39].

### 5.4. Barriers to Health Seeking Behaviours among People Who Use Drugs

The barriers to formal healthcare seeking found in this study were related to the cost of healthcare, dwindling social support, lack of knowledge, and fear of arrest. On the issue of cost as a barrier to formal healthcare seeking, the majority of the interviewees reported that they were unable to seek healthcare from the hospital due to the perceived high cost of healthcare at the formal healthcare facilities. Nevertheless, there was low patronage of NHIS among people who used the drugs. The concept of perceived barrier best explains the behaviour of the respondents not visiting the hospital mainly because they think the hospital charges more than they can afford. This finding was similar to the findings of other studies which documented cost as a barrier to healthcare seeking among respondents [31, 40, 41].

The study found the dwindling nature of social support to be a barrier to health seeking as well. It is a wide held thought that the concept of the extended family system is prevalent in Ghana and for that matter; it offers a degree of social support for family members in need. With that assumption, one would expect a person who uses drugs to get support from family members to enable them to access healthcare. However, the findings of this study showed a diminished social support, thereby serving as a barrier to healthcare seeking among those who used the drugs.Consistent finding was reported in the United States where people who used drugs were not provided with social support to boost their chances of seeking care [42].

The study also found that lack of knowledge of the medical condition and appropriate place to seek healthcare was a barrier to seeking formal healthcare for ill-health. This could possibly be due to the low level of public health education on specific health conditions and appropriate health-seeking sources among the interviewees. This finding on lack of knowledge as a barrier to formal health seeking was similar to findings in other studies where knowledge was found to be a barrier to formal healthcare seeking [40, 43, 44].

Perceived discrimination was also found to be a barrier to formal health seeking among them. Some of the interviewees were scared of being arrested when they report to the hospital when ill. Probably, this could be due to the current laws in Ghana where drug use is considered as a criminal justice issue. This finding was similar to that of other studies in which discrimination was found to be a barrier to formal health seeking among the participants [43, 45].

### 5.5. Limitations

The researchers were unable to confirm the health challenges reported by the interviewees by conducting the necessary laboratory tests to know the exact medical conditions due to the study design. Also, the choice of the theoretical proposition (HBM) is an individualistic model and limited the conclusion of the study given the broader social and structural barriers the interviewees reported.

## 6. Conclusion and Implications for Policy and Practice

This study has revealed facilitators and barriers to health seeking among people who use drugs in the Sunyani Municipality. The study showed that multiple sources of healthcare were accessed by people who use drugs. Several factors also influenced their health-seeking behaviour. Policy makers, health practitioners, and stakeholders in the healthcare milieu should intensify public health education targeted at people who use drugs to help improve on their health-seeking behaviours and access to orthodox healthcare in the country and other settings with similar challenges. Interventions should be put in place to address the issue of the cost of healthcare serving as a barrier. Moreover, withdrawal and drug dependence treatment services should be integrated in the primary healthcare services provided. Basically, there is the need for a holistic care to be provided for people who use drugs.

## Figures and Tables

**Table 1:** Sociodemographic characteristics of people who use drugs.

Characteristics	Number of participants	
	Network of people who use drugs	Hospital
Age		
Below 25	1	0
25-34	2	2
35-44	5	3
45 and above	6	1
Sex		
Male	12	6
Female	2	0
Marital status		
Single	8	2
Married	1	2
Divorced	5	2
Educational level		
No formal education	1	3
Primary	1	1
JHS/SHS	12	1
Tertiary	0	1
Occupation		
Trader	2	2
Craftsmanship	3	2
Transport industry	0	1
Unemployed	9	1
Residential status		
Own house	0	1
Renting	1	0
Family house	8	5
Homeless/sleep in ghetto	5	0
Religion		
Christianity	9	3
Islam	1	2
None	4	1
Years of drug use		
Below 10	4	1
10 to 20	4	5
21 and above	6	0
Health insurance		
Subscribed	2	4
Not subscribed	12	2
**Key informant**	1	1

## Data Availability

Data for the study can be found in the study as excerpts. Full data will be available from the corresponding author upon reasonable request.
